# EEG-Informed fMRI: A Review of Data Analysis Methods

**DOI:** 10.3389/fnhum.2018.00029

**Published:** 2018-02-06

**Authors:** Rodolfo Abreu, Alberto Leal, Patrícia Figueiredo

**Affiliations:** ^1^ISR-Lisboa/LARSyS and Department of Bioengineering, Instituto Superior Técnico – Universidade de Lisboa, Lisbon, Portugal; ^2^Department of Neurophysiology, Centro Hospitalar Psiquiátrico de Lisboa, Lisbon, Portugal

**Keywords:** simultaneous EEG-fMRI, data quality, neurovascular coupling

## Abstract

The simultaneous acquisition of electroencephalography (EEG) with functional magnetic resonance imaging (fMRI) is a very promising non-invasive technique for the study of human brain function. Despite continuous improvements, it remains a challenging technique, and a standard methodology for data analysis is yet to be established. Here we review the methodologies that are currently available to address the challenges at each step of the data analysis pipeline. We start by surveying methods for pre-processing both EEG and fMRI data. On the EEG side, we focus on the correction for several MR-induced artifacts, particularly the gradient and pulse artifacts, as well as other sources of EEG artifacts. On the fMRI side, we consider image artifacts induced by the presence of EEG hardware inside the MR scanner, and the contamination of the fMRI signal by physiological noise of non-neuronal origin, including a review of several approaches to model and remove it. We then provide an overview of the approaches specifically employed for the integration of EEG and fMRI when using EEG to predict the blood oxygenation level dependent (BOLD) fMRI signal, the so-called EEG-informed fMRI integration strategy, the most commonly used strategy in EEG-fMRI research. Finally, we systematically review methods used for the extraction of EEG features reflecting neuronal phenomena of interest.

## Introduction

*Electroencephalography* (EEG) is by far the most commonly used technique to study brain function. Its millisecond temporal resolution allows the adequate sampling of the rapidly changing electrical dynamics of neuronal populations. The power spectrum of scalp EEG signals follows approximately 1/*f*^β^ power law distributions, characteristic of most scale-free dynamics found in nature. In addition, power peaks reflecting rhythmic activity, or brain oscillations at specific frequencies, can be superimposed. In general, the most relevant brain oscillations are found in the following conventional frequency bands: delta (0.5–4 Hz), theta (4–8 Hz), alpha (8–13 Hz), beta (13–30 Hz), and gamma (above 30 Hz) (Niedermeyer and Lopes Da Silva, 2005; Akay, 2006). Both rhythmic and arrhythmic ongoing wide-band EEG activities can be modulated by external stimuli or tasks (e.g., cognitive, somatosensory, motor, auditory, visual), inducing time-locked and/or phase-locked activation of specific neuronal populations. In both cases, stimuli- or task-specific electrical potentials – *event-related potentials* (ERPs) – are generated.

Scalp EEG signals result from the mixture of propagating electric potential fluctuations (the so-called *local field potentials*, LFPs), mainly reflecting the postsynaptic activity of large populations of cortical pyramidal cells. These cells display a geometric configuration and an orientation in relation to the skull that are favorable to the constructive summation of the associated electrical current sources (Michel et al., 2004). Modest contributions from *multi-unit activity* (MUA) associated with action potentials, as well as from glial cells, have also been reported (Nunez and Silberstein, 2000; Dale and Halgren, 2001). Unfortunately, EEG source reconstruction is a notably ill-posed inverse problem, yielding a non-unique solution for all admissible potential distributions, because the number of sources is typically much larger than the number of sensors (Michel and Murray, 2012). Available strategies for the practical resolution of this problem have been proposed, but generally provide limited spatial resolution and a heterogeneous spatial sensitivity particularly for superficial versus deep sources.

Alternatively, *blood oxygenation level dependent* (BOLD) *functional magnetic resonance imaging* (fMRI) (Ogawa et al., 1990; Belliveau et al., 1991; Kwong et al., 1992) can be used to map brain activity with excellent spatial localization power, based on neurovascular coupling mechanisms producing hemodynamic changes associated with neuronal activity. Although these are not yet completely understood, both feedforward and feedback pathways have been identified, which attempt to respond to the increased demands for oxygen and glucose of brain cells. The net increase in blood oxygenation upon brain activation leads to a bulk increase in the BOLD signal (Jezzard and Toosy, 2005), due to magnetic susceptibility differences induced by the varying concentration of paramagnetic deoxyhemoglobin relative to diamagnetic oxyhemoglobin. The temporal resolution of fMRI is limited by the relatively slow hemodynamic response, with BOLD changes being delayed by several seconds relative to the onset of neuronal activity (Logothetis et al., 2001; Lewin, 2003). Usually, whole-brain fMRI measurements are performed with a spatial resolution of a few millimeters and a temporal resolution of a few seconds. Nevertheless, a trade-off between spatial and temporal resolution is possible by manipulating the image acquisition parameters; this is ultimately limited only by the available *signal-to-noise ratio* (SNR), which increases with the magnetic field strength. The use of ultrahigh field strengths (such as 7 Tesla) and highly accelerated image acquisition sequences (such as simultaneous multi-slice techniques) now allow sub-millimeter spatial resolution and whole-brain coverage in under half a second (Feinberg and Setsompop, 2013; van der Zwaag et al., 2016).

Since EEG and fMRI are the two most commonly used noninvasive functional neuroimaging techniques, and because they exhibit highly complementary characteristics, their multimodal integration has been actively sought (Laufs, 2012; Jorge et al., 2014; Murta et al., 2015). It was originally motivated by the need to accurately and non-invasively map epileptic networks in patients with drug-resistant focal epilepsy undergoing pre-surgical evaluation (Ives et al., 1993; Lemieux et al., 2001; Gotman et al., 2006; LeVan and Gotman, 2009; Gotman and Pittau, 2011; Murta et al., 2012), and it was soon extended into studies of normal brain function as well. Subject safety was addressed in early studies (Lemieux et al., 1997) and the consequent hardware modifications of the EEG recording apparatus (Goldman et al., 2000; Vasios et al., 2006) were very effective at preventing any relevant side effects in the large number of recordings performed up to this date. Moreover, increasingly efficient signal processing tools have been developed for removing the MR-induced EEG artifacts (Allen et al., 1998, 2000; Niazy et al., 2005). Although to a lesser extent, attention has also been devoted to the BOLD signal distortions caused by the presence of EEG materials (Krakow et al., 2000; Stevens et al., 2007; Mullinger et al., 2008a). Modality-specific artifacts are also present, which may further confound simultaneous EEG-fMRI analyses: while eye movements and blinks, muscle activity and bad channels are typically captured by EEG (Chaumon et al., 2015), BOLD-fMRI signals are contaminated by fluctuations of non-neuronal origin (Birn, 2012; Murphy et al., 2013). Motivated by the greater sensitivity and spatial resolution/specificity of *intracranial EEG* (icEEG), simultaneous icEEG-fMRI recordings can now also be performed (Vulliemoz et al., 2011), after both safety (Carmichael et al., 2010) and data quality (Carmichael et al., 2012) concerns have been addressed. Compared with scalp EEG, icEEG can capture more subtle and local features of electrophysiological activity, and may therefore offer novel insights into the relationship of such features with concurrent BOLD signal changes.

**FIGURE 1:**
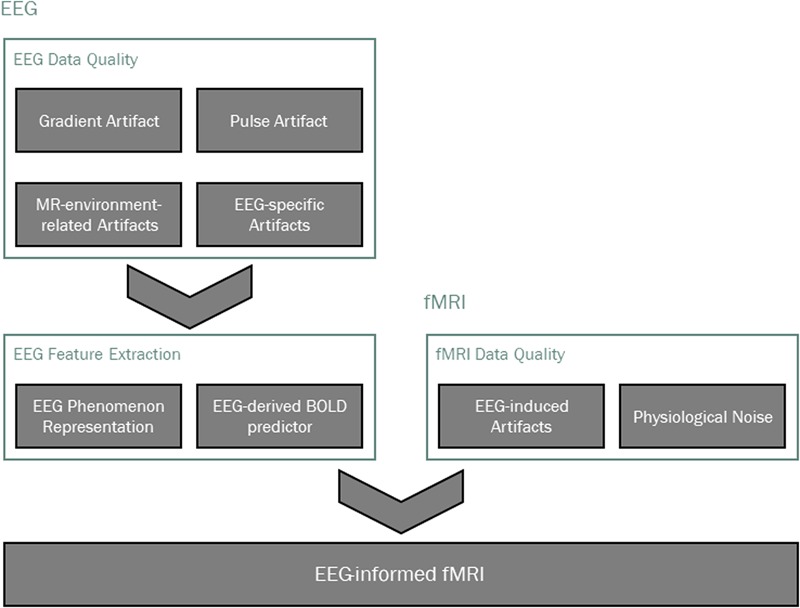
Main processing pipeline steps in EEG-informed fMRI analysis. Data quality is typically addressed first, taking into account modality-specific artifacts, as well as those that are induced by one technique on the other. The EEG phenomenon of interest is then identified and appropriate features are extracted, from which a BOLD signal predictor is derived for the localization of EEG-related BOLD-fMRI changes.

When combining EEG and fMRI, an integration strategy must be chosen. Symmetrical approaches are in principle ideal to make the most of the multimodal information, since they do not constrain any of the specific modalities, potentially creating biased estimations. These can be roughly divided into model-based and data-driven EEG-fMRI fusion techniques. While the former present great challenges namely regarding model inversion (Valdes-Sosa et al., 2009), the latter have been mostly based on *independent component analysis* (ICA) and *canonical correlation analysis* (CCA) [for reviews, refer to Rosa et al. (2010a), Lei et al. (2012)]. Information theory approaches have also been employed for the integration of EEG-fMRI data, based on the study of neuronal population codes explicitly taking into account the experimentally observed stimulus-response signal probability distributions [for reviews, refer to Panzeri et al. (2008), Ostwald and Bagshaw (2011)]. In the context of EEG-fMRI, such approaches allow the quantitative evaluation of the amount of information embodied in EEG and fMRI features (separately and jointly), to determine which features are more discriminative of the brain activity under study, and the extent to which such information overlaps across the two modalities (Ostwald et al., 2010, 2011, 2012). Because of their relative conceptual and methodological simplicity, asymmetrical approaches are by far the most common (Rosa et al., 2010a). These comprise: (1) fMRI-driven EEG, whereby the activated brain regions identified with fMRI are used as spatial constraints for the EEG source reconstruction problem (for a recent review on this topic, refer to Lei et al., 2015); and (2) EEG-informed fMRI, whereby the brain activity recorded with EEG is used to predict hemodynamic changes measured with fMRI (Gotman et al., 2006; Mulert and Lemieux, 2009; Gotman and Pittau, 2011; Laufs, 2012; Jorge et al., 2014; Murta et al., 2015). The main processing pipeline steps of EEG-correlated fMRI analyses in general are illustrated in **Figure 1**. Regardless of the integration strategy, the process of going from the first step (data acquisition) to the final result (a brain activity/connectivity map) is always confronted with several challenges.

In this review, we cover the most important data analysis steps in EEG-informed fMRI. We start by considering the issues regarding the data quality of both the EEG and the BOLD recordings, together with the respective artifact correction techniques. We then overview the multiple approaches used for EEG-informed fMRI analyses, which rely on extracting appropriate features from the EEG data in order to derive a predictor of the BOLD signal associated with the brain activity under study.

## EEG Data Quality

In this section, we provide a comprehensive characterization of multiple MR-induced EEG artifacts, as well as the respective correction techniques. The most important EEG artifacts are the *gradient artifact* (GA) and *pulse artifact* (PA), as well as head motion artifacts; other artifact sources have also been reported in the MR-environment, including the Helium cooling pump, the patient ventilation system and the room lights. In general, artifact correction techniques can be subdivided into three main types. First, the most commonly used approach consists of the time-domain subtraction of artifact templates. Typically, a template is generated for each artifact occurrence by averaging across neighboring occurrences, assuming that the artifact changes slowly over time. Second, *blind source separation* (BSS) techniques, particularly ICA, have also been used to separate EEG artifact sources from neuronal sources. In temporal ICA, an *N × M* EEG dataset, with *N* channels and *M* time points, is decomposed into a linear combination of *L independent components* (ICs) with *N × L* weights (Bell and Sejnowski, 1995; Lee et al., 1999). By removing artifact sources when back-reconstructing the EEG data to its original space, an artifact-free EEG is obtained, which makes the identification of artifact-related ICs crucial for accurate EEG cleaning. Third, some approaches rely on dedicated hardware to measure the artifact waveforms directly, followed by their subtraction from the artifact-contaminated EEG signal.

In the next two sections, we focus on the two most important MR-induced EEG artifacts, the GA and the PA, by first characterizing them and then surveying the methods used for their correction; these are summarized in **Tables 1** and **2**, respectively. In the third section, other MR-environment-related artifacts are also presented and discussed, as well as EEG-specific artifacts originating from sources unrelated with the concurrent acquisition of MR images.

**Table 1:** List of GA correction methods. These methods can be roughly divided into four main approaches: filtering, AAS-based (AAS and its several variations), ICA-based (as well as IVA, an extension of ICA to multiple datasets) and hardware-based.

Type of approach	Brief description	Reference
**Filtering**	Temporal filtering to remove GA frequency band	Hoffmann et al., 2000; Grouiller et al., 2007

**AAS**	AAS (Original IAR)	Allen et al., 2000
	AAS + PCA (on residuals)	Niazy et al., 2005
	PCA	Negishi et al., 2004
	Weighted AAS	Ritter et al., 2007; Freyer et al., 2009
	AAS + Derivatives (Taylor’s expansion)	Wan et al., 2006a
	Clustering (to group artifact occurrences) + AAS	de Munck et al., 2013
	fMRI motion parameters (to group artifact occurrences) + AAS	Moosmann et al., 2009

**ICA**	Correlation between ICs and GA template	Grouiller et al., 2007
	Use of ICA without precise synchronization between EEG and MRI systems	Ryali et al., 2009
	Independent vector analysis (IVA)	Acharjee et al., 2015

**Hardware-based**	Reference layer artifact subtraction (RLAS)	Chowdhury et al., 2014
	Prospective motion correction (PMC; camera-tracker)	Maziero et al., 2016

**Table 2:** List of PA correction methods. These methods can be roughly divided into four main approaches: AAS-based (AAS and OBS), ICA-based (with selection and removal of PA-related ICs or combination with AAS/OBS), hardware-based and others.

Type of approach	Sub-type	Brief description	Reference
**AAS**		Original AAS	Allen et al., 2000
		OBS	Niazy et al., 2005

**ICA**	IC selection and removal (based on:)	Correlation with ECG or PA templates	Srivastava et al., 2005; Mantini et al., 2007a
		Auto-correlation function	Deburchgraeve et al., 2008
		Spectral content	Vanderperren et al., 2007
		Peak-to-peak values	Vanderperren et al., 2010
		Variance explained	Debener et al., 2008
	Combination of ICA and AAS/OBS	OBS + IC removal	Debener et al., 2005, 2007
		IC removal + OBS on remaining ICs	Liu et al., 2012
		ICA + AAS/OBS on selected ICs	Abreu et al., 2016b

**Hardware-based**		Piezoelectric motion sensors	Bonmassar et al., 2002
		Loops of carbon-fiber wire	Masterton et al., 2007
		Subset of insulated electrodes to capture artifacts	Xia et al., 2014a,b; Jorge et al., 2015b
		Prospective motion correction (PMC) using camera-tracker	LeVan et al., 2013

**Other**		PA estimation from EEG signal	Krishnaswamy et al., 2016

### Gradient Artifact

#### Characterization

**FIGURE 2:**
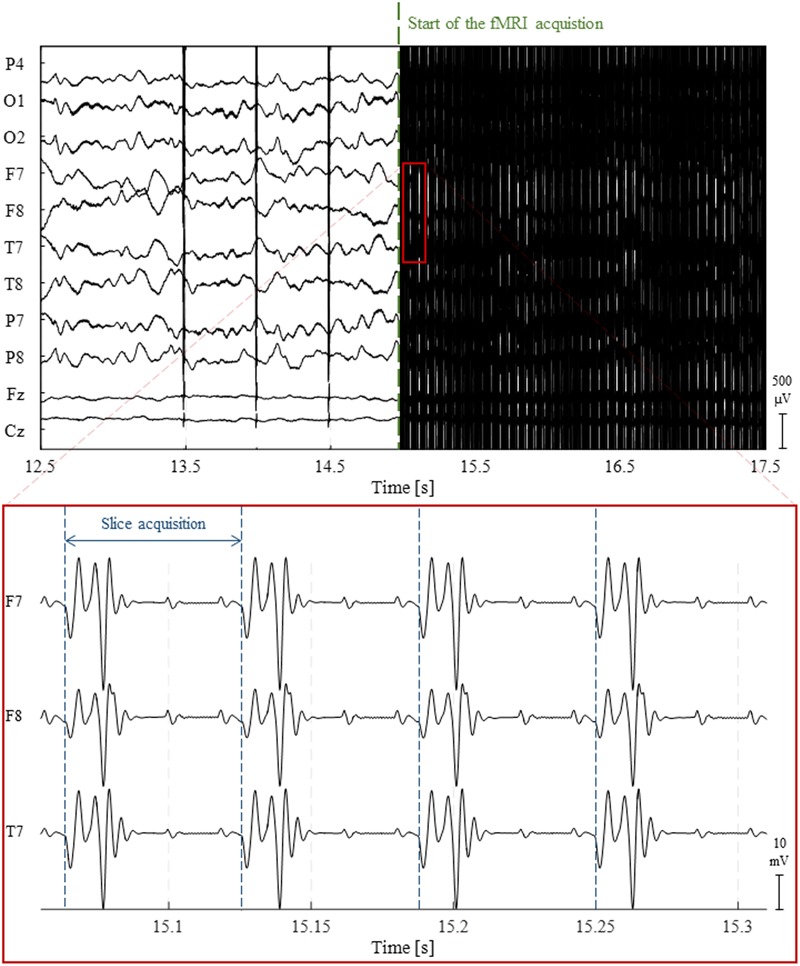
Illustration of the gradient artifact (GA) generated by a 2D multi-slice EPI sequence. (Top) 5 s traces of raw EEG data from 10 channels. At approximately 15 s, the fMRI acquisition starts, completely obscuring any neuronal activity being recorded. (Bottom) The zoomed red box shows the high-amplitude electrical potentials generated by the time-varying gradients applied during the acquisition of four image slices using 2D multi-slice EPI; due to their clear periodicity and precise timing, these artifacts can be accurately corrected using channel-specific average template subtraction techniques.

During fMRI acquisitions, the magnetic field inside the scanner changes over time due to the application of time-varying magnetic field gradients (Allen et al., 2000; Niazy et al., 2005). According to Faraday’s law of induction, these will induce an electromotive force within the conducting loop formed by the subject’s head and the EEG hardware (electrodes, wires and amplification system). A spurious voltage is hence generated on the EEG electrodes which is usually called GA or imaging artifact (Grouiller et al., 2007). The GA waveform generated by a commonly used 2D multi-slice *echo-planar imaging* (EPI) sequence for fMRI acquisition at 3 Tesla is displayed in **Figure 2**. The amplitude of such artifact can be one hundred times greater than that of the physiological EEG signal. More importantly, its spectral content usually overlaps with frequency bands of interest of the EEG, making its removal resorting to basic filtering strategies generally inappropriate. It should be noted, however, that in a few cases, MR sequences can be designed to induce artifacts at nonessential EEG frequencies, hence allowing a Fourier domain correction (Hoffmann et al., 2000; Grouiller et al., 2007). One study employed electromagnetic theory to develop a physical model for the GA (Yan et al., 2009). This model allowed the derivation of the optimal head orientation and position inside the MRI scanner in subsequent experiments, which was shown to significantly minimize the impact of MR gradients on the EEG recordings (Mullinger et al., 2011).

#### AAS Approaches

Due to its strong deterministic component, most of the methods found in the literature for GA removal follow an *average artifact template subtraction* (AAS) approach known as the *image artifact reduction* (IAR) method (Allen et al., 2000), whereby artifact templates are derived for each occurrence by averaging across multiple fMRI slice or volume epochs. Prior to averaging, a high-pass filtering is typically applied (< 1 Hz) to remove slow artifact amplitude modulations; alternatively, these can be modeled as a low-frequency sinusoidal wave (Grouiller et al., 2007). An *adaptive noise cancellation* (ANC) filter is then usually applied to remove residual artifacts. Additionally, *principal component analysis* (PCA) can be performed prior to ANC, in order to capture the residual variance within a limited number of *principal components* (PCs), which are then linearly fitted to, and subtracted from, each residual occurrence. Alternatively, PCA can also be applied directly to the EEG data (Negishi et al., 2004), followed by a similar procedure to IAR to correct for residual artifacts (Niazy et al., 2005). Although artifact templates can be defined based on the periods of either the imaging slice or volume, a combination of both slice and volume artifact template subtraction has been found to yield superior artifact correction, particularly at frequencies in the gamma band (Gonçalves et al., 2007).

Despite the remarkable precision and reproducibility of the GA, the accuracy of artifact template computation, and consequently the efficiency of average artifact subtraction, are heavily dependent on a precise synchronization between the EEG and fMRI acquisitions (Allen et al., 2000; Niazy et al., 2005). If not synchronized, a high-precision alignment of each artifact occurrence to a given reference period may still be obtained by up-sampling the EEG signal (Allen et al., 2000; Ritter et al., 2007). Ultimately, MR sequence timing parameters, namely the volume and slice repetition times as well as the time delay between the acquisition of the first volume and respective slice, can be estimated at the original EEG sampling rate prior to artifact correction (Gonçalves et al., 2007; Koskinen and Vartiainen, 2009). However, this time alignment model assumes that artifact occurrences are highly reproducible, which is only guaranteed if electrode motion is negligible.

Electrode motion is mainly due to the subjects’ head movement and system vibrations (Yan et al., 2010), and it introduces additional variance in the GA over time (Mullinger and Bowtell, 2011). For the artifact template to capture such variance, several variations of the IAR method were proposed in the literature. In particular, the artifact template can be obtained by weighting each occurrence based on its time difference from (Ritter et al., 2007), or spectral similarity to (Freyer et al., 2009), the occurrence to be corrected for. Alternatively, artifact occurrences can be clustered based on similarity measures, and an artifact template is then computed for each cluster (de Munck et al., 2013). Artifact occurrences can also be modeled as a linear combination of an artifact template and its derivatives in a truncated Taylor’s series expansion (Wan et al., 2006a). Alternatively, head motion can be estimated from the fMRI time series, and the resulting realignment parameters used to group artifact occurrences based on the degree of motion contamination; group-specific artifact templates are then derived (Moosmann et al., 2009).

#### ICA Approaches

The use of ICA for GA correction poses the challenge of selecting the associated ICs. For example, ICs can be probed for artifact-related waveforms by appropriately thresholding the correlation coefficients between each IC and an artifact template (Grouiller et al., 2007). Importantly, ICA can be used even in the presence of timing errors. In this case, an average artifact template is first computed for each occurrence and randomly time-jittered within a reasonable range. The resulting templates are then subjected to ICA, and the timing errors are expected to be reflected in the resulting ICs (Ryali et al., 2009). An extension of ICA for multiple datasets – *independent vector analysis* (IVA) – has also been proposed for GA correction (Acharjee et al., 2015). In this method, EEG data are first segmented using the fMRI scanning triggers as time-locking events, and the multiple segmented channels are then entered as multiple datasets for IVA. The resulting IVA components are maximally dependent across channels, and maximally independent across segments for a given channel. GA sources are thus separated from those of different origins, as in ICA, but also taking into account spatial dependencies of GA waveforms across electrodes, and hence allowing for a more accurate, channel-wise GA source estimation (Acharjee et al., 2015).

#### Hardware-Based Approaches

New EEG caps have been developed, incorporating a second set of EEG electrodes that overlay those in contact with the scalp, separated by a reference layer (Chowdhury et al., 2014). While the electrodes located below the reference layer are physically attached to the scalp as in a standard EEG cap, capturing a mixture of artifacts and brain signals, the electrodes located above the reference layer are electrically isolated from the scalp so that they do not pick up brain signals and hence measure only MR-induced artifacts. These include not only the GA, but also the PA, as well as motion-driven artifacts. By taking the difference between the signals measured by the scalp electrodes and the corresponding reference electrodes, an artifact-free EEG signal can be obtained.

In a different approach, an MR-compatible camera-tracker device is attached to the subject’s head in order to monitor head motion. *Prospective motion correction* (PMC) of the EEG signal can then be performed based on the head translation and rotation parameters estimated along the three main axes. These estimates are typically used to improve MR image quality, by updating the specifications of the RF pulses and MR gradients during the image acquisition in real-time (recent reviews on these approaches can be found in Maclaren et al., 2013; Zaitsev et al., 2016). In the work by Maziero et al. (2016), these motion parameters are additionally used to model, and subsequently regress out, the motion-induced voltages on the concurrently acquired EEG.

#### Methods Comparison

At least two comparative studies of multiple GA correction methods can be found in the literature (Grouiller et al., 2007; Ritter et al., 2007). By taking into account not only the extent of artifact removal, but also the associated degradation of the EEG signal of interest, both studies arrived at the conclusion that the performance of each method is highly dependent on the type of EEG activity of interest, namely in terms of its frequency band. Appropriate balancing between artifact and physiological signal removal can be achieved by directly comparing performance measures between scan and non-scan periods (Freyer et al., 2009). This requires the acquisition of artifact-free EEG data during MR-silent periods, which can be obtained using a dedicated EPI sequence characterized by a long *repetition time* (TR; 4070 ms) (Anami et al., 2003). Ritter et al. (2007) investigated the impact on the GA correction algorithms of using different parameters (namely the number of artifact windows to build the template, and whether PCA should be employed to remove residual artifacts or not), and found sets of optimal parameter values for each GA correction method.

In our experience, the deterministic component of GA renders the use of the original IAR method suitable for most applications, avoiding the challenging selection of GA-related ICs in ICA-based methods, or the introduction of additional hardware to the typically intricate EEG-fMRI setups. Whenever facing unsatisfactory results, one can start by varying the number of artifact occurrences used to generate the artifact template, and thus differently weighting the amount of artifact reduction (lower number of occurrences) relative to the preservation of EEG physiological signal (higher number of occurrences). Variations of the original IAR can also be tested; however, in our experience these variations were only successful at capturing GA variability at the slice level and lack sensitivity at the volume level.

### Pulse Artifact

#### Characterization

The PA, also commonly referred to as the ballistocardiogram artifact, is currently one of the most challenging artifacts in the EEG acquired concurrently with fMRI, mainly due to its non-stationary nature determined by the temporal variability of the cardiac pulse. As discussed in Yan et al. (2010) and Mullinger et al. (2013), several mechanisms contribute to the PA by inducing voltage changes on the scalp EEG electrodes due to interactions with the strong, static magnetic field of the MRI scanner, namely: (1) bulk head rotation due to cardiac blood ejection (Bonmassar et al., 2002); (2) scalp expansion due to arterial pulsation (Debener et al., 2008); and (3) Hall effect caused by the pulsatile flow of the blood, which is an electrically conductive fluid (Tenforde et al., 1983). One study found that most of the artifact variance was explained by flow-induced Hall voltage and pulse-driven head rotation (Mullinger et al., 2013). Importantly, the PA amplitude significant increases with the static magnetic field strength, *B*_0_ (Debener et al., 2008; Neuner et al., 2013), such that it can severely hamper the visual inspection of typical EEG behaviors at 7 Tesla (Jorge et al., 2015a,b).

#### AAS Approaches

**FIGURE 3:**
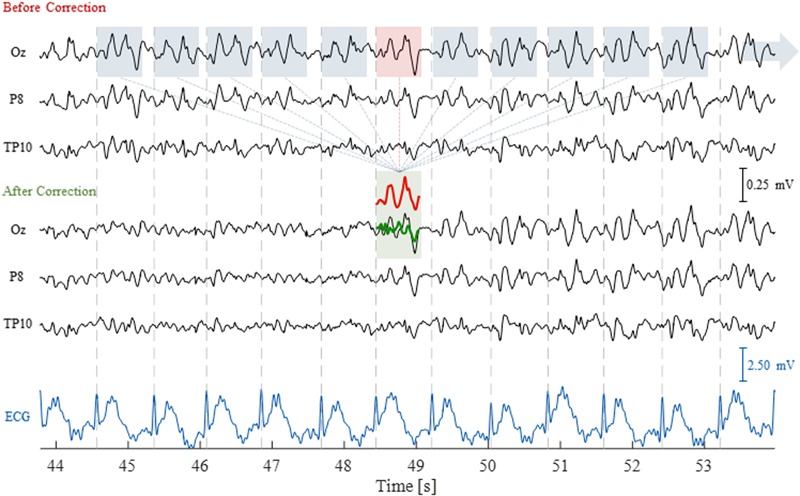
Illustration of the AAS technique to correct for the pulse artifact (PA). The EEG traces are shown for three channels, before and after correction, as well as for the ECG channel, over a time period of 10 s including 11 artifact occurrences. The segmented windows for each artifact occurrence (blue and red boxes) were averaged to compute the artifact template (red trace); this was then subtracted from the corresponding artifact occurrence (red box), yielding the artifact-corrected signal (green trace).

Because the PA is roughly periodic, with artifact occurrences being approximately time-locked with the cardiac cycle, an AAS algorithm can be employed, similarly to the case of the GA. In this case, an artifact template is extracted from the EEG signal by averaging across multiple cardiac cycles, followed by a time-domain subtraction procedure (illustrated in **Figure 3**) (Allen et al., 1998). Unlike the GA, however, the much greater variability over time must be taken into account for effectively reducing the PA. This can be achieved by computing the temporal PCA over all time-locked occurrences of the artifact in order to build an *optimal basis set* (OBS), comprising a given number of PCs that explain the PA variance to some extent (Niazy et al., 2005); this basis set is then fitted to, and subtracted from, each artifact occurrence.

#### ICA Approaches

If ICA can accurately separate the PA sources from other sources contributing to the EEG signal, a PA-free EEG signal may be obtained by reconstructing the EEG without the PA-related ICs (Bénar et al., 2003; Srivastava et al., 2005; Mantini et al., 2007a). However, the objective and accurate classification of PA-related ICs remains a major concern, and several criteria can be found in the literature for that purpose; these are reviewed in Vanderperren et al. (2010) and their performance is compared in Abreu et al. (2016b). Briefly, such criteria can be roughly divided into five main types, whereby PA-related ICs are identified by: (1) thresholding the correlation coefficients between each IC and the simultaneously acquired ECG signal (Mantini et al., 2007a), or a PA template (Srivastava et al., 2005); (2) probing the auto-correlation function of each IC for peaks located at the distance between two consecutive QRS peaks (Deburchgraeve et al., 2008); (3) identifying ICs that exhibit spectral peaks at cardiac-related frequencies, exploring the periodic nature of the PA (Vanderperren et al., 2007); (4) thresholding the *peak-to-peak* (PTP) values of the back-projected QRS-triggered EEG data when using each IC individually, under the hypothesis that PA-related ICs should exhibit higher PTP values (Vanderperren et al., 2010); and (5) thresholding the amount of artifact variance that is explained by each source (Debener et al., 2008). Following the rationale of the latter, we have recently proposed the PROJIC (*PROJection onto Independent Components*) algorithm, whereby the average PA waveform of each channel is first projected onto the IC space by means of the corresponding un-mixing matrix estimated using ICA. The power of each projection, computed as the squared sum for each time instant, is then clustered by *k*-means; ICs assigned to high-powered clusters are deemed PA-related (Abreu et al., 2016a).

Due to the relative advantages and disadvantages of AAS- and ICA-based methods, their combination has been proposed (Debener et al., 2005, 2007). In one method, the OBS method is first used to remove most of the PA contribution from the EEG data and ICA is then employed to further remove residual artifacts by excluding PA-related ICs from the back-reconstruction of the EEG signal. The use of a modified version of OBS in the IC space instead has also been proposed, whereby non-PA-related ICs are corrected for the PA, while PA-related ICs are removed (Liu et al., 2012). Such procedure was motivated by the hypothesis that the artifact contributes to all ICs in varying degrees due to its non-stationary nature. Following a similar rationale, we have recently proposed the application of AAS and OBS in the IC space, but only to PA-related ICs, which are subsequently kept in the EEG signal reconstruction together with the original non-PA-related ICs (Abreu et al., 2016a). This approach aims to preserve the physiological signal as much as possible without compromising artifact removal efficiency.

#### Hardware-Based Approaches

Motion sensors can be used to monitor pulse-driven as well as voluntary head movements during concurrent EEG-fMRI acquisitions; the sensor signals can be used to estimate head motion and associated EEG artifacts, which can then be subtracted from the EEG data (Bonmassar et al., 2002). Different kinds of motion sensors have been used for reducing the PA in simultaneous EEG-fMRI recordings. PA waveforms can be obtained by means of loops of carbon-fiber wire that are physically attached to, but electrically isolated from, the subject’s head (Masterton et al., 2007). More recently, a simple modification of a standard EEG cap has been proposed, whereby four of the existing electrodes are insulated from the scalp and directly connected to the reference electrode so that they can be used as motion sensors (Jorge et al., 2015a). PA waveforms can also be extracted from a layer of electrodes added to a standard EEG cap and separated from the scalp by two insulating layers (Xia et al., 2014a,b). A practical approach in this case consists of defining a set of insulated electrodes spatially surrounding each uninsulated electrode, in order to build an accurate estimate of the local PA (Xia et al., 2014b). Following this rationale, complex methods to determine the optimal minimum number of electrodes to insulate have been developed based on the spatial redundancy of PA measured from neighboring electrodes (Xia et al., 2014a). In an alternative approach, an MR-compatible camera-tracker device has also been shown to improve PA correction, by first converting the measured six motion parameters into velocities, and then modeling the PA-induced EEG voltages as a linear combination of the low-pass filtered velocities (LeVan et al., 2013). In a similar approach, which, however, avoids additional hardware in order to minimize the complexity of the simultaneous EEG-fMRI setup, the PA is estimated directly from the EEG as a linear combination of several cardiac-related harmonics (Krishnaswamy et al., 2016).

#### Methods Comparison

Simultaneous EEG-fMRI measurements of event-related activity are typically used for assessing the performance of a given correction method, based on metrics computed from the ERPs such as: the inter-trial variability (Vanderperren et al., 2010), the SNR (Debener et al., 2007), and the difference between the ERPs extracted from the inside-MR EEG datasets and those that are obtained from the PA-free outside-MR EEG data (Mantini et al., 2007a). When the frequency content of an event-related EEG signal is known, the power within that frequency band can also be computed before and after PA correction (Xia et al., 2014a). In *resting-state* EEG-fMRI (rs-fMRI) studies, the PA occurrences can be compared before and after correction based on their *root mean square* (RMS) or PTP values (Chowdhury et al., 2014). Additionally, the total spectral power within windows around the cardiac fundamental frequency and its first harmonics can be computed, in order to quantify the amount of PA that is removed (Liu et al., 2012). Similarly to the evaluation pipeline described in Freyer et al. (2009) for the GA, we have proposed to assess the trade-off between PA and physiological signal reductions by computing ratios of the power over specific frequency bands and then linearly combining them using a weighting factor that describes the importance given to the preservation of physiological signal relative to artifact correction (Abreu et al., 2016a).

Grouiller et al. (2007) found that AAS was the method of choice if highly accurate QRS detection was achieved. Additionally, and as discussed in Vanderperren et al. (2010), OBS and ICA-based approaches only yielded comparable results if the ICA parameters were fine-tuned. In fact, optimizing the algorithms’ parameters has been shown to critically affect the efficiency and reliability of the subsequent analyses (Vanderperren et al., 2010; Abreu et al., 2016a), particularly at high magnetic field strengths (Debener et al., 2008). According to our own study (Abreu et al., 2016a), AAS was the method exhibiting the second best results in terms of accurately removing the artifact while preserving the physiological signal of interest. In contrast, purely ICA-based methods either resulted in substantial residual artifacts, or significantly distorted the physiological signal. The best results were obtained by combining ICA to separate the PA sources, with AAS to correct the artifact occurrences in the IC space. We believe that this may be a simple but effective solution for PA correction, which does not require additional hardware.

### Other Sources of EEG Artifacts

#### MR-Environment-Related Artifacts

Several artifacts are induced on the EEG in the MR environment, even without an ongoing fMRI acquisition. Typically, these artifacts are caused by electrode motion as a result of MR scanner vibrations associated with the Helium compression pumps used for cooling down MR components, the patient ventilation system, and the room lights (Mullinger et al., 2008a; Mulert and Lemieux, 2009; Nierhaus et al., 2013; Neuner et al., 2014; Rothlübbers et al., 2014). In particular, the Helium pump artifact has been characterized in some systems by prominent peaks in the EEG spectrum around frequencies of 50 and 100 Hz (Rothlübbers et al., 2014). The ventilation system and room lights are reflected in spectral peaks at other specific frequencies, with the former depending on the ventilation level (Nierhaus et al., 2013). While turning off the room lights does not present any clear compromise, switching off the ventilation system may cause patient discomfort. In principle, the Helium pump cooling system can also be turned off; however, this may carry the associated risk of Helium boil-off in certain systems (Mullinger et al., 2008a) and it is not permitted in some clinical sites for safety and procedural reasons. Due to its repetitive nature, the Helium pump artifact can be adequately removed by employing AAS-based approaches (Rothlübbers et al., 2014). Alternatively, PCA has been recursively applied to EEG segments in order to separate the components associated with the Helium pump artifact; components exhibiting a single peak within a frequency range typically spanning the artifact were removed from the data (Kim et al., 2015).

#### EEG-Specific Artifacts

Although not caused by the MR environment, other sources of artifacts that contaminate the EEG must also be accounted for in simultaneous EEG-fMRI studies, namely: eye movements, saccades and blinks, muscle activity, and bad channels (Chaumon et al., 2015). Eye movements are usually picked up by frontal electrodes, although artifactual waveforms may also be observed on distant electrodes (Urigüen and Garcia-Zapirain, 2015). The degree of contamination by this type of artifact is determined by the proximity of the electrodes to the eyes, as well as the direction of the movement (Croft and Barry, 2000). Eye saccades can be either horizontal or vertical, both mainly captured by frontal electrodes, and are characterized by abrupt changes in the EEG amplitude. These changes can be mistaken with EEG activity in the gamma band (Yuval-Greenberg et al., 2008). Eye blinking is also more prominent in frontal electrodes, usually inducing high-frequency artifacts due to its abrupt nature (Croft and Barry, 2000; Urigüen and Garcia-Zapirain, 2015). The EEG topographies associated with eye blinks resemble those of vertical eye saccades (Chaumon et al., 2015). Muscle artifacts result from the myogenic activity caused by contracting muscles, particularly those surrounding the mandible, and their effects depend on the degree of contraction and the type of muscle (Goncharova et al., 2003). Muscle artifacts usually span a wide frequency band of the EEG, although they considerably overlap with beta activity (∼15–30 Hz) (Goncharova et al., 2003; McMenamin et al., 2010). This type of artifacts can also affect a large cortical surface area due to volume conduction of myogenic activity from different head muscles (Urigüen and Garcia-Zapirain, 2015). The so-called bad channels are usually associated with high-impedance electrodes and are typically characterized by strong fluctuations that are uncorrelated with the remaining electrodes (Chaumon et al., 2015).

Some of these types of EEG artifacts are expected to be mainly stationary, making the use of ICA particularly suitable. For this purpose, techniques have been proposed for the automatic selection of the artifactual ICs, which are then removed from the back-reconstruction of the EEG data (for reviews, refer to Vanderperren et al., 2010; Chaumon et al., 2015; Urigüen and Garcia-Zapirain, 2015). Most of those techniques, however, rely on artifact-specific *a priori* information, rendering them unsuitable for other types of EEG activity (Campos Viola et al., 2009; Nolan et al., 2010; Mognon et al., 2011; Daly et al., 2014; Chaumon et al., 2015).

## MR Data Quality

In this section, we start by briefly characterizing MR image artifacts that are induced by the presence of EEG hardware inside the MR scanner. Although unrelated with EEG, signal fluctuations of non-neuronal origin are known to contaminate BOLD-fMRI data, especially in resting-state studies of spontaneous activity. For this reason, such fluctuations are also considered here, including their characterization and an overview of the methods used for their modeling and removal.

### EEG-Induced Image Quality Degradation

The presence of EEG hardware during MR image acquisitions is known to degrade image quality, although to a substantially lesser degree when compared with the effects of such acquisitions on the EEG signal (Mulert and Lemieux, 2009; Mullinger and Bowtell, 2011). Since MR image quality is closely related with the amplitude and homogeneity of both static (*B*_0_) and oscillating (*B*_1_) magnetic fields, and because the EEG system will directly interfere with those magnetic fields, appreciable effects on MR data quality are expected.

Firstly, magnetic susceptibility differences between the EEG system materials and the head tissues induce perturbations on *B*_0_, which in turn cause geometric distortions and signal loss in the MR images. These effects scale linearly with both the magnetic susceptibility difference and the field strength (Mullinger et al., 2008b; Jorge et al., 2015b), and depend on the spatial orientation of the material relative to *B*_0_ (Krakow et al., 2000). Secondly, the conductive materials of the EEG system, particularly leads outside the EEG cap because of the longer wires connected to them, also cause perturbations on the transmitted *B*_1_ field. These alter the effective flip angle of imaging sequences and hence induce local image intensity variations, which are exacerbated if *B*_0_ homogeneity is also compromised (Mullinger et al., 2008b). Additionally, *B*_1_ field shielding also occurs due to loop currents induced on the electrically conductive materials of the EEG electrodes and leads (Stevens et al., 2007), which is aggravated when using high-density EEG caps (Mullinger and Bowtell, 2011). Overall, these effects of the EEG on *B*_1_ lead to image SNR losses, and also to local changes in the *specific absorption rate* (SAR) of MR image acquisition sequences (Vasios et al., 2006; Stevens et al., 2007). The latter scale linearly with the number of electrodes and the *B*_0_ field strength (Angelone et al., 2006). The use of specifically designed low-SAR sequences is therefore crucial to ensure subject safety and comfort in simultaneous EEG-fMRI recordings at ultra-high fields (Nöth et al., 2012).

The electromagnetic noise generated by the EEG recording components can be minimized through appropriate shielding and the use of suitable materials, which should be as diamagnetic as possible (Krakow et al., 2000; Stevens et al., 2007). However, modifications of the electrodes are limited: on the one hand, they must not compromise the overall functionality of the EEG system; on the other hand, they must ensure subject safety, namely by including current-limiting resistors (Lemieux et al., 1997). Moreover, only a minimal quantity of conductive gel should be used, so that it does not induce appreciable image artifacts while still providing acceptable electrode impedance (Krakow et al., 2000). Novel EEG caps have been developed using different technologies in order to minimize the impact of simultaneous EEG-fMRI recordings on subject safety and MR data quality (Vasios et al., 2006), and ultimately to make feasible the recording of high-density EEG data at ultra-high magnetic field strengths (Poulsen et al., 2017).

In addition, *B*_0_ and *B*_1_ inhomogeneities can be accounted for through post-processing based on *B*_0_ and *B*_1_ field maps obtained using dedicated MR sequences (Ericsson et al., 1995; Yarnykh, 2007). Although increasingly faster field mapping sequences are being developed (Eggenschwiler et al., 2012), the associated additional scanning time may not be available in conventional imaging settings. Nevertheless, current literature suggests that negligible effects are observed on image quality in humans when using commercially available, low-density EEG caps at field strengths of 3 Tesla (Mullinger et al., 2008b). At 7 Tesla, however, important *B*_1_ field distortions have been reported (Jorge et al., 2015b). Most importantly, although the spatial SNR is significantly affected, the temporal SNR (tSNR) of fMRI seems to be relatively preserved, in part because physiological noise is also reduced with the overall signal loss; in fact, no significant differences have been reported when comparing BOLD-fMRI detection sensitivity with or without the EEG cap in place (Luo and Glover, 2012; Klein et al., 2015).

### BOLD-fMRI Physiological Noise

#### Characterization

BOLD-fMRI signal changes result from contributions from both neuronal and non-neuronal origins. The latter include cardiac and respiratory sources commonly referred to as physiological noise (Hutton et al., 2011; Brooks et al., 2013; Caballero-Gaudes and Reynolds, 2017). The arterial pulsation associated with cardiac function produces brain tissue movements, as well as changes in *cerebral blood volume* (CBV) and *cerebral blood flow* (CBF), across the cardiac cycle (Purdon and Weisskoff, 1998; Krüger and Glover, 2001; Greitz et al., 2010). As for respiration, the thoracic modulation within each respiratory cycle produces bulk head motion, as well as changes in *B*_0_ (Raj et al., 2001) and in the arterial CO_2_ partial pressure (Wise et al., 2004). These changes associated with the cardiac and respiratory cycles induce correlated, quasi-periodic BOLD fluctuations, located predominantly near and within large blood vessels and more generally across the brain, respectively (Birn, 2012). The flow of *cerebrospinal fluid* (CSF) is also modulated by both cardiac and respiratory cycles, resulting in associated BOLD signal changes in CSF-filled regions (Klose et al., 2000). Bulk motion related with the cardiac and respiratory cycles leads to confounds similar to the ones produced by voluntary head motion, even with the use of head restraints (Murphy et al., 2013). Typically, they manifest as correlated signal changes at the edges of the brain and in regions with large spatial variations in image contrast. Additionally, non-periodic BOLD signal fluctuations are also produced due to changes in cardiac rate (Shmueli et al., 2007), as well as in breathing depth and rate leading to changes in the CO_2_ arterial partial pressure (Birn et al., 2006).

**FIGURE 4:**
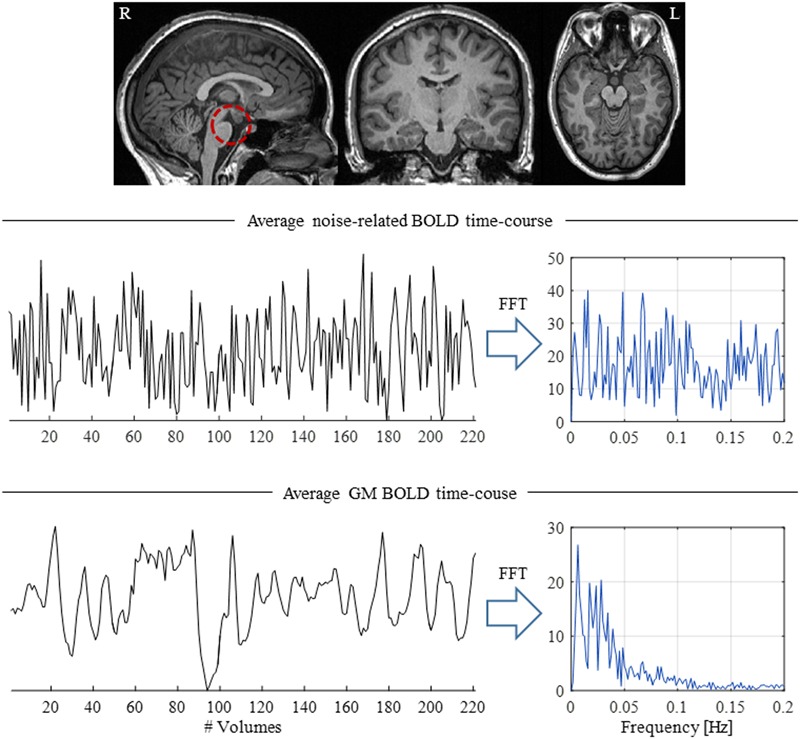
Illustration of the presence of physiological-related fluctuations in the BOLD signal. (Top) Structural brain image highlighting the brainstem (red dashed circle), a brain structure located close to major arteries and CSF-filled spaces, thus particularly susceptible to physiological fluctuations. (Middle) Average physiological noise-related BOLD time-course and respective power spectrum computed from a region near the brainstem. (Bottom) Average BOLD time-course across GM; in contrast with the brainstem, the GM time-course presents a clear 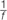 spectrum, with most of its power located at frequencies below 0.1 Hz.

These physiologically driven effects, if left uncorrected, may compromise the analysis of fMRI data, particularly when studying spontaneous activity (Biswal et al., 1995; Cordes et al., 2001; Birn, 2012; Murphy et al., 2013), and they are therefore a concern also in simultaneous EEG-fMRI (Liston et al., 2006; van Houdt et al., 2009; Abreu et al., 2017b) (**Figure 4**). Since a typical fMRI acquisition sequence uses a TR of ∼2–3 s, aliasing of both cardiac (≈ 1 Hz) and respiratory (≈ 0.3 Hz) fundamental frequencies will inevitably occur (Bhattacharyya and Lowe, 2004), making the use of temporal filtering strategies unsuitable unless a very short TR < 0.4 s is used (Biswal et al., 1996). Such a short TR would allow only partial brain coverage with conventional sequences. However, faster sequences have recently been developed, allowing whole-brain coverage with such short TR values (Feinberg and Setsompop, 2013), which opens up new possibilities for physiological noise characterization and correction. A number of physiological noise correction approaches have been proposed, including both model-based (relying on external physiological recordings and/or the fMRI data itself) and data-driven techniques; these are reviewed next, and summarized in **Table 3**.

**Table 3:** List of BOLD-fMRI physiological noise correction methods. These methods can be roughly divided into four main approaches: filtering, physiological recordings-based, image-based and data-driven.

Type of approach	Brief description		Reference
**Filtering**	Using sequences with very short TR (< 0.4 s)		Biswal et al., 1996

**Physiological recordings-based**	RETROKOR		Hu et al., 1995
	RETROICOR		Glover et al., 2000
	RETROICOR + timing errors from volume registration		Jones et al., 2008
	Extended RETROICOR (RETROICOR and:)	P_ET_CO_2_	Wise et al., 2004
		RV/RVT (surrogates of P_ET_CO_2_)	Birn et al., 2006; Chang et al., 2009
		Estimation of respiration from ECG	van Houdt et al., 2009; Abreu et al., 2017b
		HR	Shmueli et al., 2007
		Cardiac and respiratory response functions (CRF/RRF)	Birn et al., 2008; Chang et al., 2009
		Lag optimization of RV/RVT and HR	Bianciardi et al., 2009a; Falahpour et al., 2013; Jorge et al., 2014

**Image-based**	CSF and WM fluctuations (estimation based on:)	Temporal standard deviation (tSTD)	Behzadi et al., 2007
		Fitting mixture of Gaussians to a robust temporal SNR (tSNR) measure	Tierney et al., 2015
		PCA on noise-related regions	Behzadi et al., 2007; Bianciardi et al., 2009b; Jorge et al., 2014; Tierney et al., 2015
	Head motion parameters (estimation based on:)	fMRI volume alignment	Murphy et al., 2013
		Navigator echoes	Thesen et al., 2000; Welch et al., 2002
		Camera-tracker devices	Zaitsev et al., 2016
		Active markers	Ooi et al., 2011
		EEG data	Zotev et al., 2012; Wong et al., 2016
	Removal of volumes highly affected by motion		Power et al., 2012; Tierney et al., 2015

**Data-driven**	ICA + manual identification of ICs		Griffanti et al., 2017
	ICA + automatic identification of ICs		De Martino et al., 2007; Tohka et al., 2008; Churchill et al., 2012b; Smith et al., 2013; Salimi-Khorshidi et al., 2014

#### Physiological Recordings-Based Approaches

An important class of physiological noise correction methods relies on recording, concurrently with the fMRI acquisition, external cardiac and respiratory signals by means of appropriate sensors, usually a plethysmograph and a respiratory belt, respectively. A retrospective correction method that works directly in *k*-space (RETROKCOR) was first introduced, aiming to remove the contribution of physiological noise prior to image reconstruction (Hu et al., 1995). However, most methods operate in the image space, and are generally extensions of the *retrospective image-based correction* (RETROICOR) method (Glover et al., 2000). The contribution of periodic cardiac and respiratory processes is described by a low-order Fourier expansion in terms of the phases of the cardiac and respiratory signals in relation to the fMRI acquisition time; several model orders have been tested, as well as their interactions (Harvey et al., 2008). These terms (cardiac, respiratory and interactions) are then estimated and regressed out from the fMRI data using a *general linear model* (GLM) framework. Since RETROICOR relies critically on the timing of image acquisition relative to each cardiac and respiratory cycle, and because such timing can be confounded by head motion, the incorporation of timing errors introduced by volume registration into the Fourier expansion of the cardiac phase at each voxel has been proposed (Jones et al., 2008).

Non-periodic, respiratory-related contributions to the BOLD signal were first tackled by measuring the end-tidal CO_2_ partial pressure (P_ET_CO_2_) using a capnograph, and including its time-course as a confounding regressor in the GLM (Wise et al., 2004). Alternatively, the *respiratory volume per unit time* (RVT) can be obtained directly from the respiratory recordings, and is commonly used as a surrogate of P_ET_CO_2_ (Birn et al., 2006). Similarly, changes in *heart rate* (HR) have also been found to induce confounding BOLD fluctuations (Shmueli et al., 2007). In order to describe the contributions of RVT and HR to the BOLD signal in a linear systems framework, a *respiratory response function* (RRF, Birn et al., 2008) and a *cardiac response function* (CRF, Chang et al., 2009) have been estimated, respectively. These are then convolved with the RVT and HR time courses, respectively, following a similar rationale to the one underlying the use of the *hemodynamic response function* (HRF) to describe the BOLD response to neuronal activity (Friston et al., 1998). Alternatively, RVT and HR regressors can be shifted by an appropriate time lag in order to maximize the BOLD signal variance explained (Bianciardi et al., 2009a; Jorge et al., 2013). In order to account for the large inter- and intra-subject variability, both RRF/CRF estimation and time lag optimization can be performed specifically for each subject or even brain region (Falahpour et al., 2013; Pinto et al., 2017). Our own (Abreu et al., 2017b) and other studies (van Houdt et al., 2009) have shown that, if only cardiac data is available, it is possible to derive the corresponding respiratory data from this, and to successfully denoise BOLD-fMRI data using the resulting surrogate of the respiratory signal.

#### Image-Based Approaches

Another method of physiological noise correction is based on the extraction of confounding regressors from the fMRI data itself (Murphy et al., 2013). Because BOLD signal fluctuations of neuronal origin should be mainly located in *gray matter* (GM), CSF and *white matter* (WM) fluctuations are likely to reflect predominantly physiological noise contributions. Typically, the average BOLD time-courses within CSF and/or WM masks are computed and regressed out from the fMRI data in a GLM framework. Variations of this approach can be found in the literature in terms of the definition of the noise-related regions, as well as the features to be extracted from them. As for the former, thresholding of voxel-specific *temporal standard deviation* (tSTD) measures can be performed, supported by the observation of a positive correlation between the variance explained by the respective RETROICOR regressors and the tSTD value for a given voxel (Behzadi et al., 2007). A biophysically inspired measure of robust tSNR has also been proposed, whereby a mixture of Gaussians is fitted to this metric in each voxel using an expectation-maximization approach (Tierney et al., 2015). Regarding the extraction of features, PCA can be applied to the BOLD signals from noise-related regions, so that the variability of the physiological fluctuations can be taken into account (Behzadi et al., 2007; Bianciardi et al., 2009b; Jorge et al., 2013; Tierney et al., 2015). The identification of the optimal number of PCs to keep is then crucial, in order to avoid under-/over-estimation of the contribution of physiological fluctuations if an excessively low/high number of PCs is included in the model, respectively (Behzadi et al., 2007).

Furthermore, head motion parameters are also commonly regressed out from the BOLD signal. These are usually extracted from the fMRI time-series in the motion correction pre-processing step, in which each brain volume in the series is aligned to a reference volume by estimating a rigid body transformation characterized by three translation and three rotation parameters, yielding a total of six motion regressors (Murphy et al., 2013). Because large, abrupt head movements are not accurately estimated using such affine transformations, metrics identifying fMRI volumes affected by this type of motion can be computed, so that these volumes may be removed from subsequent analyses (Power et al., 2012; Tierney et al., 2015). In order to improve the temporal resolution of the motion regressors, several approaches have been proposed in the literature, mostly based on the use of navigator echoes (Thesen et al., 2000; Welch et al., 2002), camera-tracker devices (Zaitsev et al., 2016), and active markers (Ooi et al., 2011). Although they do not need additional hardware unlike tracker devices and active markers, the use of navigator echoes requires longer acquisition times (a recent review on this topic can be found in Godenschweger et al., 2016). Alternatively, in simultaneous EEG-fMRI sessions, the EEG data may be used to derive such highly sampled regressors, allowing the correction of motion at the slice level retrospectively (Zotev et al., 2012; Wong et al., 2016). Regardless of the estimation procedure, special attention must be devoted to cases where head motion is correlated with task parameters of interest, seriously confounding subsequent analyses, because plausible, but yet spurious, effects may be observed (Fellner et al., 2016).

#### Data-Driven Approaches

Independent component analysis can be used for fMRI de-noising by separating sources of scanner artifact, physiological noise and brain activation (Beckmann and Smith, 2004; Brooks et al., 2008). By removing the contribution of ICs reflecting non-neuronal fluctuations from the back-reconstruction of the data, a noise-free BOLD signal is obtained. Such identification procedure is critical and it can be done manually (Griffanti et al., 2017) or by resorting to automatic classification tools, based on the temporal and spatial characteristics of the expected artifacts (De Martino et al., 2007; Tohka et al., 2008; Churchill et al., 2012b; Smith et al., 2013; Salimi-Khorshidi et al., 2014).

#### Data Pre-processing and Physiological Noise Correction

There is currently no consensus regarding the order by which the various steps in the processing pipeline of fMRI data should be taken to include physiological noise correction. In particular, the question whether RETROICOR should precede slice timing correction or not, and what the order between this and motion correction should be, remains open. One study found that RETROICOR should be performed prior to slice timing correction (Jones et al., 2008). Another recent study investigated the optimal processing pipeline more systematically, by testing the impact of 48 different combinations of the main processing steps on task-based activation maps (Churchill et al., 2012a). The combination of motion correction with a second order polynomial detrending yielded the highest performance on average, but a large variability across subjects was found for the optimal order of the processing steps, indicating that a subject-specific optimization should preferentially be carried out. In general, inter-subject variability and the need for subject-specific optimization of physiological noise correction have been reported in several studies (Falahpour et al., 2013; Golestani et al., 2015; Nunes et al., 2015), including our own (Abreu et al., 2017b).

In fact, in our study we showed the importance of removing not only cardiac and respiratory fluctuations, as well as those of WM, CSF and head motion origins, although the former explained BOLD signal variance to a much lesser extent than the latter. This claim is in line with the results and recommendations made by Jo et al. (2010), indicating that the removal of cardiac and respiratory fluctuations is crucial, particularly when subjects exhibit a high variability in HR and breathing depth and rate during the acquisitions. Another important question relates to the inclusion of additional terms in the physiological noise model. Typically, statistical testing is applied in a nested model approach to ascertain if the additional variance explained by a given term relative to that explained by those already included in the model is significant. Although an order of two is most commonly used for RETROICOR, with the frequent addition of RV/RVT, HR, WM, CSF and motion parameters, in the so-called extended RETROICOR approaches, it may be important to investigate the impact of specifically identifying which regressors to include in the model in each study.

## EEG-Informed fMRI Integration Methods

In this section, we review the data integration methods used for mapping brain networks using an EEG-informed fMRI approach. These are largely sub-divided into univariate and multivariate methods and they are summarized in **Table 4**. In univariate methods, a limited number of EEG time-courses (often a single time-course) representative of the phenomena of interest is selected, and temporal or spectral features are then extracted and used to predict BOLD changes. In contrast, multivariate methods consider multiple EEG channels in this feature extraction step. The rationale underlying the choice of method for the extraction of such features mainly depends on the type of activity of interest. Epileptic activity is particularly relevant in the scope of simultaneous EEG-fMRI studies, given the suitability of this technique for the localization of brain networks associated with epileptic discharges, and therefore extensive literature on the extraction of epilepsy-related EEG features predictive of BOLD fluctuations is available (Gotman et al., 2006; Marques et al., 2009; Mulert and Lemieux, 2009; Gotman and Pittau, 2011; Laufs, 2012; Jorge et al., 2014; Murta et al., 2015; Abreu et al., 2018).

**Table 4:** List of EEG features predictive of BOLD signal fluctuations of interest. The methods used to derive such EEG features can be roughly divided into univariate (temporal, spectral and intra-cranial features) and multivariate (spatial correlation features, functional connectivity methods and others).

Type of features	Name	Reference
**Univariate methods**		
Temporal events	Stick and boxcar functions	Lemieux et al., 2001; Bagshaw et al., 2005; Jacobs et al., 2009; Thornton et al., 2010
	IED amplitude, energy and width	Bénar et al., 2002; LeVan et al., 2010
	ERP amplitude and response latency	Debener et al., 2005; Bénar et al., 2007; Fuglø et al., 2012; Nguyen and Cunnington, 2014; Wirsich et al., 2014
	IED amplitude, width, slope of the rising phase, energy and spatial extent (intra-cranial EEG)	Murta et al., 2016
Spectral features	EEG power across frequency bands	Goldman et al., 2002; Mantini et al., 2007b; de Munck et al., 2009
	Total power	Wan et al., 2006b
	Linear combination of band-specific power values	Goense and Logothetis, 2008
	Mean frequency	Rosa et al., 2010a
	Root mean squared frequency	Kilner et al., 2005; Rosa et al., 2010a
	Phase-amplitude coupling (intra-cranial EEG)	Murta et al., 2017
**Multivariate methods**		
Spatial correlation features	Spatial template from separate EEG recordings	Grouiller et al., 2011
	EEG microstates	Britz et al., 2010; Yuan et al., 2012; Schwab et al., 2015
	Continuous ESI	Vulliemoz et al., 2010
Functional connectivity	Partial directed coherence	Biazoli et al., 2013
	Phase synchronization index	Mizuhara et al., 2005; Kottlow et al., 2012; Abreu et al., 2018
Other approaches	Multiway decomposition methods	Schwab et al., 2015; Marecek et al., 2016
	EEG channel-specific BOLD predictors	Meir-Hasson et al., 2014

### Univariate Methods

In univariate methods, a time-course (or a limited number of time-courses) representative of the phenomenon of interest must be obtained prior to the extraction of features predictive of the BOLD signal. Both temporal and spectral features are commonly extracted and will be specifically described here.

#### EEG Time-Courses Representative of Phenomena of Interest

If the phenomenon of interest of brain activity is reflected within a limited set of electrodes (e.g., posterior alpha rhythms measured by occipital electrodes), subsequent analyses can be focused on those electrodes alone (Goldman et al., 2002). However, in general, this dimensionality reduction problem is not trivial, and spatial filtering strategies must be applied to the EEG data. Assuming that fluctuations have a specific temporal structure, these can be extracted from the EEG using linear prediction algorithms that incorporate such prior knowledge about the frequency and location of the sources of interest to be isolated (Ferdowsi et al., 2015). This prior knowledge can also be incorporated in a semi blind source separation (s-BSS) technique named *Functional Source Separation* (FSS, Porcaro et al., 2010), biasing the estimation procedure toward sources comprising the physiological aspects of interest. If such information is not available, conventional BSS techniques must be employed.

Temporal ICA is the most commonly used spatial filtering strategy in this context (Marques et al., 2009; Formaggio et al., 2011; Leite et al., 2013). However, the selection of the ICs that best reflect the phenomenon of interest is crucial, and different selection criteria can be found in the literature. In a visual inspection approach, the IC topographies can be probed for spatial patterns resembling that expected for a given activity of interest (Debener et al., 2005). A more objective approach consists of identifying the ICs that exhibit the highest weights within a pre-specified set of electrodes expected to reflect the activity of interest (Scheeringa et al., 2008; Ke and Shen, 2010). The temporal dynamics of the IC time-courses can also be inspected for events of interest (Jann et al., 2008), and a number of quantitative methods have been proposed based on spectral criteria, particularly the power within a given frequency band (Formaggio et al., 2011). In studies where more than one EEG acquisition is performed, the reproducibility of the ICs across runs can also be used as a selection criterion (Leite et al., 2013).

A few studies have developed automatic methods for selecting ICs representing specific activities of interest based on the use of respective templates: *component assessment* (COMPASS) (Wessel and Ullsperger, 2011), *spatiotemporal templates for independent component selection* (STTICS) (Abreu et al., 2015), and PROJIC (Abreu et al., 2016b). The COMPASS and STTICS algorithms are based on the explicit similarity of the ICs with spatiotemporal templates of the activity of interest. Both methods assume that a single spatial map describes such activity, which is, however, not true if a given EEG source has a certain degree of non-stationarity. In contrast, and as briefly described above, PROJIC first projects a temporal template (an average EEG event) onto the IC space, and then clusters the resulting projections based on their power. In our experience, PROJIC has been found to accurately identify both epilepsy-related ICs as well as PA-related ICs (Abreu et al., 2016a,b). Such versatility arises from the fact that PROJIC is not based on the explicit similarity to spatiotemporal templates, which renders it potentially suitable in many other applications.

#### Temporal Features

In the simplest approach for combining EEG and fMRI, the EEG signal is visually inspected for events of interest. This is essentially used in epilepsy: while *inter-ictal epileptiform discharges* (IEDs) are treated as zero-duration events and modeled as stick functions, ictal activity is represented by boxcar functions (both usually referred to as unitary regressors) (Lemieux et al., 2001; Bagshaw et al., 2005; Jacobs et al., 2009; LeVan and Gotman, 2009; Leal et al., 2016). A finer representation of the seizure dynamics can be achieved by dividing these epileptic events into a succession of stages (e.g., early ictal, clinical seizure onset and late ictal), and modeling each stage by a separate boxcar function (Thornton et al., 2010). In the case of IEDs, the amplitude of the stick functions can be modulated by different IED features, namely their amplitude, energy or width, which has been found to improve the correlation with BOLD signal changes (Bénar et al., 2002; LeVan et al., 2010). When recording icEEG, the amplitude, width, slope of the rising phase, energy and spatial extent of IEDs are possible predictors of epilepsy-related BOLD changes; in a recent study, only the width was found to explain additional variance to that of unitary regressors (Murta et al., 2016).

More generally, in stimuli/task-based EEG-fMRI studies, features extracted from the associated ERPs on a trial-by-trial basis can also be used to predict BOLD fluctuations, namely the trial-specific amplitude and response latency (Debener et al., 2005; Bénar et al., 2007; Fuglø et al., 2012; Nguyen and Cunnington, 2014; Wirsich et al., 2014).

#### Spectral Features

In order to account for the rich temporal and spectral profiles of the EEG, more complex transfer functions between the EEG and BOLD signals have been proposed based on time-frequency decompositions of the EEG signal (Goldman et al., 2002; Moosmann et al., 2003; Laufs et al., 2006; Scheeringa et al., 2008). One of the first studies using spectral features mapped EEG alpha fluctuations by extracting the average EEG power within the alpha band across four occipital channels, at epochs of the same duration as the repetition time of the fMRI acquisition sequence (Goldman et al., 2002). A similar procedure can be applied to other EEG rhythms. By including the EEG power over multiple frequency bands as regressors in a GLM analysis of the fMRI data, their individual contributions to the BOLD signal, as well as their interactions, can be investigated (Mantini et al., 2007b; de Munck et al., 2009). Besides the EEG power across specific frequency bands, several other features of the spectrogram have also been proposed to explain the BOLD signal, namely: total power (Wan et al., 2006b), linear combination of band-specific power values (Goense and Logothetis, 2008), mean frequency (Rosa et al., 2010b), and *root mean squared frequency* (RMSF) (Kilner et al., 2005; Rosa et al., 2010b). Studies comparing the predictive power of these features in healthy subjects (Rosa et al., 2010b), and in an epilepsy case study (Leite et al., 2013), found that RMSF outperformed other power-weighted metrics, in agreement with the heuristic model proposed by Kilner et al. (2005). In icEEG recordings, the single-trial phase-amplitude coupling strength when performing a motor task has been found to explain BOLD variance in addition to that from a combination of EEG power across several frequency bands (Murta et al., 2017).

### Multivariate Methods

Multivariate methods use data from multiple EEG channels in order to capture spatial information, which cannot be assessed by univariate approaches. They include methods based on the spatial correlation of the EEG with reference spatial maps, functional connectivity measures across different EEG channels, as well as other multiway decomposition methods.

#### Spatial Correlation Methods

In resting-state studies of healthy volunteers, BOLD correlates of EEG microstates have been investigated, based on the hypothesis that resting-state networks are reflected in both signals. Predictors of spontaneous BOLD fluctuations occurring during rest have been obtained, by spatially correlating the concurrent EEG topographies at each time point with the previously identified EEG microstates (Britz et al., 2010; Yuan et al., 2012). Such EEG microstates can be derived by clustering (Britz et al., 2010), ICA (Yuan et al., 2012), or topographic time-frequency decomposition (Schwab et al., 2015). The latter combines knowledge from techniques for time-domain spatial analysis of EEG and time-frequency decomposition of single time-courses (Koenig et al., 2001).

In a similar approach, one study proposed to use epilepsy-specific spatial templates derived from separate, long-term EEG recordings of epileptic activity (Grouiller et al., 2011). They found that the spatial correlation between these templates and the EEG scalp topographies measured at each time point during simultaneous EEG-fMRI recordings provided a good BOLD predictor. This approach may be advantageous in cases where it is not possible to detect epileptic events on the EEG recorded simultaneously with fMRI.

In a somewhat related approach, local estimates of electrical activity can be obtained by *electrical source imaging* (ESI). This tool estimates the location of EEG sources in the brain responsible for generating a given topography measured at the scalp. Considering a topography representative of epileptic activity, Vulliemoz et al. (2010) determined the averaged current density within the ESI solution for the whole EEG (continuous ESI), and used it to predict the associated local BOLD changes. One study showed that this approach yielded a more accurate epileptic network mapping when compared to that described in Grouiller et al. (2011), providing concordant electro-clinical localization of the epileptic focus in all investigated patients (Elshoff et al., 2012).

#### Functional Connectivity Methods

Other multivariate EEG measures have been employed, particularly with the purpose of reflecting functional connectivity across the brain. In one study, the partial directed coherence (a directed measure of functional connectivity) across different frequency bands has been correlated with BOLD, in order to map the intra- and inter-hemispheric flow of information measured with EEG (Biazoli et al., 2013). Most interestingly, EEG phase synchronization measures can also be used, with the advantage that they do not depend on the amplitude of the EEG signal, in contrast with most temporal and spectral features, which renders them less susceptible to artifacts. However, such measures have been scarcely used as predictors of BOLD. In particular, Mizuhara et al. (2005) successfully mapped task-dependent BOLD signal changes using the *phase synchronization index* (PSI) computed for a specific frequency and channel pair. A global connectivity measure has also been employed, *global field synchronization* (GFS), which quantifies the overall EEG synchrony across the scalp. In a task-based EEG-fMRI study, Kottlow et al. (2012) used GFS to predict BOLD changes associated with face integration. In a resting-state study, GFS measures within the lower (8.5–10.5 Hz) and upper (10.5–12.5 Hz) alpha band were found to be positively correlated with the BOLD-fMRI-derived dorsal attention network and the default mode network, respectively (Jann et al., 2009). More recently, a study by our group applied both PSI and GFS in an EEG-fMRI study of epilepsy (Abreu et al., 2018). We showed that PSI within a frequency band of interest outperformed power-weighted metrics, as well as GFS, in predicting BOLD changes associated with epileptic activity. More importantly, we showed that PSI more specifically reflects epileptic activity, rather than motion-related spurious signal changes. We believe that the potential use of EEG synchronization measures for predicting BOLD fluctuations of interest should thus be further explored in different applications.

#### Other Multivariate Methods

By applying multiway decomposition methods, the multi-dimensional EEG spectrum can be blindly decomposed into patterns characterized by spatial, spectral and temporal signatures. The use of these methods allowed the mapping of thalamic substructures associated with scalp EEG signals (Schwab et al., 2015), as well as task-related BOLD signal changes more accurately than commonly used power-weighted EEG features (Marecek et al., 2016).

In a different approach, Meir-Hasson et al. (2014) tested the accuracy of different combinations of features (frequency band and time delay) extracted from each channel to predict BOLD fluctuations of interest in the visual cortex. In each iteration, the prediction error is used as feedback to guide the next combination of features to be tested; upon convergence, an optimal EEG-derived model of BOLD is obtained for each channel, and thus avoiding the spatial filtering step.

### Other Integration Approaches

Although a GLM framework is by far the most commonly used for the integration of EEG and fMRI, alternative methods have also been proposed in order to overcome assumptions regarding the shape of the HRF, the linearity between EEG activity and the BOLD signal, and the probability distribution of noise in the data (e.g., Caballero-Gaudes et al., 2013). Even within the GLM framework, several approaches have been proposed to account for variable HRF shapes, and hence improve the sensitivity of detecting BOLD changes. A popular method consists of allowing variations around the canonical HRF, by adding its temporal and dispersion derivatives to the GLM, in order to account for deviations in both time-to-peak and time of onset (Friston et al., 1998). More flexible methods can be used by considering Fourier or finite basis sets (Goutte et al., 2000; Thornton et al., 2010). Ultimately, the shape of the HRF can be estimated freely as a finite impulse response for each voxel separately (Glover, 1999; Lu et al., 2006; de Munck et al., 2007; Storti et al., 2013). One study employed a set of gamma-based HRFs in which the two main parameters (time-to-peak and time of onset) were systematically varied, in order to choose the best suited to model the HRF in epilepsy patients (Grouiller et al., 2010). Subject- and pathology-specific HRFs can also be defined by estimating combinations of multiple HRFs peaking at different latencies (Bagshaw et al., 2005). Nonetheless, studies assessing the minimal degree of spatial specificity (e.g., voxel versus brain regions) that is required in order to accurately map BOLD fluctuations of interest without the risk of overfitting are still missing.

Most EEG-informed fMRI studies focus on predicting the BOLD signal measured at each voxel based on the EEG. However, new opportunities for EEG-informed fMRI have recently been created by the growing interest in the study of the temporal fluctuations of BOLD functional connectivity across the brain, the so-called *dynamic functional connectivity* (dFC) (Hutchison et al., 2013; Calhoun et al., 2014; Preti et al., 2016). In fact, a number of studies have already attempted to incorporate EEG data in dFC analyses, which are reviewed in Tagliazucchi and Laufs (2015). Normal brain function was first investigated by correlating EEG fluctuations of interest (mainly band-specific EEG power) with dFC fluctuations, in order to identify electrophysiological correlates of functional connectivity patterns (Scheeringa et al., 2012; Tagliazucchi et al., 2012; Chang et al., 2013; Allen et al., 2017). The relevance of using EEG to inform analyses of BOLD dFC fluctuations in epilepsy is also starting to be investigated, with promising results regarding the identification of epileptic networks that are no longer assumed to be static over time, as in standard EEG-informed fMRI studies as those described in the previous sections (Preti et al., 2014; Abreu et al., 2017a). Although the field of dFC is relatively recent and further studies are needed to better understand the physiological meaning of functional connectivity fluctuations, the inclusion of EEG in dFC analyses appears to be a promising avenue to achieve such goal, opening new lines of EEG-fMRI research.

## Conclusion

In this review, we overviewed the several challenges associated with each step of the data analysis pipeline in EEG-informed fMRI, and provided a comprehensive description and discussion of the plethora of methods available to address each of those challenges. The motivation underlying the concurrent, multimodal acquisition of EEG and fMRI was first highlighted, including a brief description of the fundamentals of each neuroimaging modality. Special attention was then given to the critical problems concerning EEG and MRI data quality, by characterizing the artifacts induced by each modality on the other, as well as the most important modality-specific artifacts, and describing the respective artifact reduction techniques. Finally, we focused on multimodal data integration in the context of the EEG-informed fMRI approach, surveying both univariate and multivariate methods used to extract EEG features that may predict BOLD signal changes. This review may help the identification of the processing pipeline that best fits each study, in order to optimize data quality as well as the sensitivity and specificity of the brain networks obtained by EEG-informed fMRI analysis. The optimal method for the integration of data from the two modalities remains an open question, mainly because a deeper understanding about the substrates of each modality and the extent to which these substrates overlap is still needed. Furthermore, more extensive, critical and independent validation studies are needed to guide the interpretation of the findings obtained using the methods described above.

## Author Contributions

RA was responsible for reviewing the literature on simultaneous EEG-fMRI methods, and writing the review itself. AL provided crucial feedback on the more clinical aspects of the review. PF supervised the literature review and the organization of the manuscript, and participated in writing the manuscript.

## Conflict of Interest Statement

The authors declare that the research was conducted in the absence of any commercial or financial relationships that could be construed as a potential conflict of interest.
